# Digital Surveillance After Allogeneic Hematopoietic Stem Cell Transplantation Guides Therapeutic Interventions to Reduce Non‐Relapse Mortality

**DOI:** 10.1111/ejh.70159

**Published:** 2026-03-18

**Authors:** Lara Bischof, Katharina Egger‐Heidrich, Martin Schneider, Gregor Friedrich, Anna Massow, Johanna Vogelsang, Franziska Schmidt, Matthias Hänel, Thomas Illmer, Lynn Leppla, Alexandra Teynor, Sabina de Geest, Gabriele Muelller, Klaus H. Metzeler, Martin Bornhäuser, Uwe Platzbecker, Jan Moritz Middeke, Vladan Vučinić

**Affiliations:** ^1^ Department for Hematology, Cellular Therapy, Hemostaseology and Infectious Diseases University of Leipzig Medical Center Leipzig Germany; ^2^ Cancer Center Central Germany–CCCG Leipzig Germany; ^3^ Department of Internal Medicine I University Hospital Carl Gustav Carus, Technical University Dresden Dresden Germany; ^4^ Clinical Trials Unit, DKMS Group Dresden Germany; ^5^ Department of Internal Medicine III, Hospital Chemnitz Chemnitz Germany; ^6^ Private Practice Hematology and Oncology Dresden Germany; ^7^ Department of Medicine I, Medical Center ‐ University of Freiburg Faculty of Medicine, University of Freiburg Freiburg im Breisgau Germany; ^8^ Institute of Nursing Science, Department Public Health University of Basel Basel Switzerland; ^9^ Institute for Agile Software Development, Technical University of Applied Sciences Augsburg Augsburg Germany; ^10^ Academic Centre for Nursing and Midwifery, Department of Primary Care and Public Health KU Leuven Leuven Belgium; ^11^ Center for Evidence‐Based Healthcare Faculty of Medicine and University Hospital Carl Gustav Carus, TUD Dresden University of Technology Dresden Germany; ^12^ Fraunhofer Institute for Celltherapy and Immunology Leipzig Germany

**Keywords:** acute myeloid leukemia, allogeneic HSCT, digital surveillance

## Abstract

**Introduction:**

Allogeneic hematopoietic stem cell transplantation (HSCT) offers curative potential for many hematological malignancies; however, outcomes may be adversely affected by infections and transplant‐associated complications, contributing to non‐relapse mortality (NRM). Innovative digital approaches for outpatient surveillance may support earlier detection of complications and thereby help reduce NRM.

**Case Presentation:**

We report on a 73 year‐old Caucasian patient who underwent allogeneic HSCT for acute myeloid leukemia in complete remission following non‐myeloablative conditioning. The patient participated in the interventional study “Cross‐sectoral care for patients with hematological diseases following innovative cell therapy” (SPIZ), which evaluates digital surveillance after cellular therapies. SPIZ comprises daily remote monitoring of vital signs, medication adherence, and symptoms using a multimodal approach integrating an eHealth system (patient smartphone app and caregiver monitoring dashboard), home visits, video consultations, and regular case conferences with referring physicians during follow‐up. Eight months post‐HSCT, during an inpatient stay at an orthopedic rehabilitation center, the patient reported increasing dyspnea and cough via the app. In response, the SPIZ team initiated immediate transfer to the transplant center. Diagnostic work‐up revealed pneumonia caused by *aspergillus fumigatus* and coronavirus, and progression of pre‐existing pulmonary graft‐versus‐host disease (GvHD), confirmed by computed tomography and spiroergometry. Early detection and prompt transfer enabled rapid initiation of antifungal therapy and intensified GvHD management. The patient was discharged with improved general condition and respiratory function, without further septic complications.

**Conclusion:**

Innovative digital surveillance is an effective tool for outpatient monitoring after cellular therapies, facilitating early detection and intervention and potentially reducing NRM, particularly in high‐risk patients.

## 1 Introduction

Allogeneic hematopoietic stem cell transplantation (HSCT) offers the chance of cure for many patients diagnosed with high‐risk hematologic diseases [1, 2, 3]. However, it is associated with unique and common side effects, which can severely impact non‐relapse mortality (NRM) and survival. Among the most relevant contributors to NRM are infectious complications under immunosuppression, as well as transplant‐specific complications such as graft‐versus‐host disease (GvHD) [4].

To optimize the outpatient follow‐up care of patients after allogeneic HSCT and chimeric antigen receptor T (CAR‐T) cell therapy, an innovative prospective study‐control project called “Cross‐sector care for patients with hematological diseases following innovative cell therapy” (German abbreviation: SPIZ) was implemented in three German centers for cellular therapy and is currently enrolling patients. Among other components, SPIZ includes digital documentation of vital signs and symptoms. The SMILe Software “(SteM Cell TransplantatIon faciLitated by eHealth)” (SMILe) allows remote patient surveillance of patient well‐being, four medical parameters (i.e., blood‐pressure, heart rate, weight, and temperature), symptoms (intestinal: e.g., nausea, vomiting, diarrhoea, oral ulcera, loss of appetite; skin: exanthema; infection: e.g., dyssuria, cough, shortness of breath; neurotoxicity: e.g., Immune Effector Cell Encephalopathy Score, headache, tremors) and medication adherence via the SMILe app installed on patients´ smartphones. Patients can also send comments or additional questions via encrypted messages to the associated monitoring platform SMILeCare located and supervised at the transplant center [5]. App entries are monitored daily. In case of abnormalities the responsible physician is informed and may order diagnostic or therapeutic procedures or schedule a visit to address emerging issues, with goal of prevention or rapid intervention in case of infections or transplant‐specific side effects. There is no automatic or artificial inteliigence reply incorporated.

Besides enhanced observation and structured algorithms, SPIZ also offers other strategies to improve survival and quality of life in a heavily pretreated cohort. The project reduces patient visits to the transplant centers by implementing video calls with physicians and home visits of trained nurses with optional interventions such as intravenous fluid substitution, which is especially beneficial for patients with remote residence. Digital documentation of vital signs and symptoms, accessible for both referring and treating physicians, and scheduled conferences with referring physicians and associated health care workers close gaps in information transfer by improving cross‐sectoral communication, also facilitating access to social work and psychological infrastructure.

SPIZ's main goal is the reduction of NRM after allogeneic HSCT or CAR‐T cell therapy by early detection and timely interventions in the event of impeding complications [6]. The project collects data over a follow‐up period of at least 1 year, with disease‐specific rehospitalization‐free survival as a primary endpoint, and complications and quality of life as secondary endpoints. Quality of life is assessed by standardized questionnaires at predefined timepoints. Furthermore, a subgroup of patients will be analyzed for health‐insurance costs.

Here, we report on a 73 year‐old Caucasian patient, diagnosed with acute myeloid leukemia (AML). The remote app monitoring during his orthopedic rehabilitation allowed early detection of infection‐related symptoms and a worsening pulmonary GvHD, supporting rapid transfer to the transplant center and prompt therapeutic intervention.

## 2 Case Presentation

The patient received an allogeneic HSCT from a female matched unrelated donor in August 2024 for adverse risk AML according to the European LeukemiaNet 2022 classification [2]. He attained first complete remission with incomplete peripheral recovery prior to allogeneic HSCT. The patient underwent a non‐myeloablative conditioning consisting of 2 Gy total‐body irradiation and fludarabine at a total dose of 90 mg/m^2^ [7, 8]. Immunosuppression consisted of cyclosporine A, sirolimus, and mycophenolate‐mofetil [9]. Before discharge after allogeneic HSCT, the patient consented to participation in the SPIZ project.

Three months after allogeneic HSCT, bone marrow assessment detected measurable residual disease (MRD) indicative of persisting leukemic clones. Therefore, immunosuppression was expeditedly reduced with the intention to promote a graft‐vs‐leukemia effect [10, 11, 12, 13, 14]. Three months later, a complete molecular remission of the AML was documented. At the same time, the patient first presented with symptoms of pulmonary GvHD, i.e., dyspnea and reduction in walking distance to 500 m. Body plethysmography showed reduced forced expiratory volume in first second (FEV1) (64.01%, see Table 1), and computed tomography (CT) scan revealed pulmonary consolidations and bronchiolitis obliterans, both strongly suggestive for pulmonary GvHD. Furthermore, the patient developed mild dryness of ocular and oral mucosa as further potential GvHD manifestations, corresponding to National Institutes of Health grade II chronic GvHD. Additional treatment with prednisolone (1 mg/kg) and increased cyclosporine A trough levels (target 100 μg/L) resulted in rapid symptom improvement, normalization of FEV₁ (105.1%), and regression of CT abnormalities 3 months after GvHD diagnosis (see Table 1).

**TABLE 1:** Lung function results of the patient during follow‐up after allogeneic HSCT.

Time point	Lung function by spiroergometry and body pletismography				
	FEV1 [%]	FVC [%]	FEV1%FVC [%]	DLCBOcSB [%]	VC [%]
2 months before HSCT	92.4	99.6	92.4	53.8	104.4
2 months after HSCT	104.5	103.1	101.0	67.1	104.1
6 months after HSCT (first signs of GvHD)	64.0	74.4	85.7	55.6	81.2
9 months after HSCT (prior to rehabilitation)	105.1	100.2	104.4	72.0	102.5
10 months after HSCT (aspergillus pneumonia)	49	56.8	86.8	45.8	57.6
11 months after HSCT (after pneumonia)	127.7	109.3	116.3	73.1	116.0

*Note:* Spiroergometry and body pletismography results at and following allogeneic HSCT.

Abbreviations: DLCOcSB, Diffusion capacity of the lung for carbon monoxide with single‐breath method; FEV1, forced expiratory volume in the first second; FEV1%FVC, Tiffeneau index, Percentage of expiratory volume of vital capacity expired in the first second after forced exhalation; FVC, forced vital capacity; VC, vital capacity.

Seven months after allogeneic HSCT, the patient sustained a traumatic femoral neck fracture requiring surgery. Two months later, he underwent inpatient orthopedic rehabilitation. During the orthopedic hospitalization, increasing dyspnea and dry cough were reported via the SMILe app, triggering SPIZ coordination algorithms. Upon interpretation of the reports, the team contacted both the patient and the rehabilitation center and completed anamnesis of the symptoms and procedures performed, which included clinical chest X‐ray and intervention with cough‐suppressants. There was no drug monitoring or dose adaptations of the immunosuppressive medication.

**FIGURE 1:**
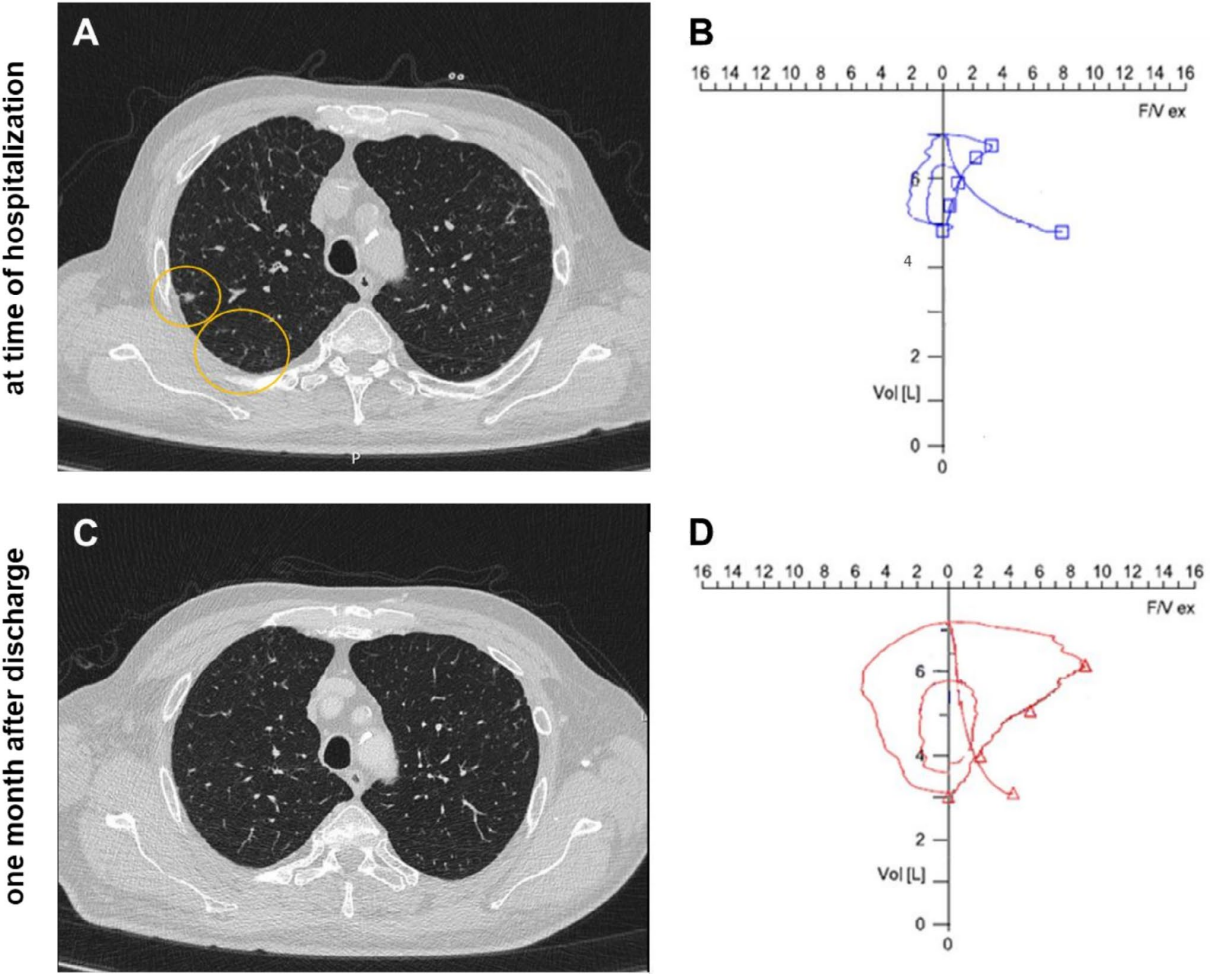
Chest CT scan and spiroergometry at SPIZ intervention and at follow‐up. Chest computed tomography scan of reported patient after transport to transplantation center and at time of detection of *aspergillus fumigatus*. Findings suggestive of fibrotic changes of the pulmonary interstitium, predominantly in the basal segments of the right upper lobe, differential diagnoses include post‐inflammatory sequelae, mycotic consolidations (marked in yellow) and pulmonary graft‐versus‐host disease (GvHD). (B) Chest computed tomography scan of reported patient at follow‐up 1 month after anti‐infective treatment. Fibrotic changes in the pulmonary interstitium. Regredient interstitial drawing in the apical and basal right upper lobe. (C) spiroergometry at time of mycotic pneumonia diagnosis and worsening of pulmonary Graft‐vs‐host‐disease. (D) spiroergometry 1 month after anti‐infective treatment.

To facilitate more extensive diagnostic evaluation regarding possible infection and/or worsening of the chronic pulmonary GvHD, we organized transport to our transplant center. On admission, he presented with exertional dyspnea (severe grade, according to Global Initiative for Chronic Obstructive Lung Disease category of Obstructive Lung Disease) and dry cough. Chest auscultation showed bilateral rhonchi, and oxygen supplementation of 2 L/min was required. Chest CT (Figure 1A+B) and bronchoalveolar lavage indentified pneumonia casued by *aspergillus fumigatus* as well as infection with endemic coronavirus. Antifungal treatment with voriconazole was initiated. Additionally, CT imaging and spiroergometry revealed intermediate obstructive lung disease consistent with bronchiolitis obliterans (see Table 1). Pulse corticosteroid therapy was initiated for suspected worsening of pulmonary GvHD. After 7 days of inpatient stay, the patient no longer required oxygen, walking distance improved to 1000 m, and he was discharged. One month after the diagnosis of pneumonia, a follow‐up CT and spiroergometry showed regression of pulmonary fibrosis and improvement of airflow obstruction (Figure 1C+D and Table 1).

## 3 Discussion

We reported on a 73‐year‐old AML patient who underwent allogeneic HSCT and received timely treatment for fungal pneumonia and pulmonary GvHD after interpretation of digital symptom reporting within an integrated care model.

Intensive surveillance at an experienced transplant center is obligatory for outpatient post‐transplantation care to detect and treat transplant‐associated adverse events without delay [15, 16, 17]. Despite advances in preemptive and prophylactic strategies, NRM remains high after allogeneic HSCT. Main contributors include infectious complications in an immunosuppressed cohort with various secondary immunodeficiencies and/or acute or chronic GvHD, which exert synergistic negative effects. Infectious complication rates reach 3.6 per patient‐year post‐HSCT [18], and impact survival especially shortly after HSCT (up to day +100) [19]. Furthermore, chronic GvHD (> day +100) occurs in 30%–40% of patients after allogeneic HSCT [20].

Multiple risk factors for chronic GvHD were present in the reported patient, including sex mismatch (female donor, male recipient) [21, 22], non‐myeloablative conditioning, advanced recipient age [10], and preemptive immunosuppression reduction for MRD addressing [23, 24]. However, strategies for reduction of immunosuppression are necessary, due to the negative prognostic impact of MRD relapse to hematological relapse as investigated by various groups [25, 26, 27, 28, 29]. Pulmonary manifestations of GvHD, as in this case, contribute to elevated NRM risk through impaired ventilation and susceptibility to opportunistic infections [30, 31]. Our patient furthermore had a history of smoking, further increasing pulmonary mortality risk [32].

This high‐risk profile highlights the importance of early diagnostics and intervention in case of complications and underscores the relevance of remote monitoring outside healthcare facilities [16, 33]. In this case, the SPIZ‐enabled intervention prevented delayed diagnosis during rehabilitation and prevented possible septic transformation and further irreversible impairment of ventilation.

Remote monitoring and home visits enable a longer stay at home, which has been shown to positively affect post‐transplantation complication rates. There is comparable data from the Swedish Karolinska Institute offering early discharge in the pancytopenic phase after non‐myeloablative allogeneic HSCT or autologous HSCT by implementing daily nurse visits rather than wait until after blood count regeneration and oral nutrition. Multiple studies of this institute performed since 1988 showed that early outpatient monitoring results in significantly lower higher‐grade GvHD, earlier weaning from intravenous parenteral nutrition, and a lower number of infections [34, 35, 36, 37, 38]. This translated into a lower 100‐day NRM of 14.1% vs. 22.6% (*p* = 0.041) for early outpatient vs. inpatient follow‐up care, respectively [38]. A strategy with reduced hospital stay may also affect mental health positively [39]. Beside SPIZ, there is a comparable pilot project in Spain called ‘My Medula’, having published data on feasibility of self‐records in the first 2 months after allogeneic HSCT of 28 patients, leading to a high degree of patient satisfaction. A randomized trial regarding cost‐effectiveness and outcome endpoints is currently enrolling [40].

As the evaluation of endpoints is pending, we briefly comment on the feasibility of implementing such a system and its limitations. One of the main prerequisites is patient adherence, as well as the ability to use digital devices and digital health information, particularly in an elderly cohort. Additionally, the geographic radius of residence must be limited (200 km for this project) around the study center. The costs of home visits will be evaluated in a centralized analysis after study completion. Previously reported data from implementation trials of the SMILe eHealth‐facilitated integrated care model, albeit with limited participant numbers, have demonstrated benefits in terms of survival, rehospitalization‐free survival, and cost‐effectiveness [41]. Implementation costs include training and licensing fees for nurses, case managers, referring physicians, and on‐case physicians, as well as medical training and transportation costs. Currently, the project focuses on early post‐cellular therapy care (1 year), but future expansion is conceivable.

To our knowledge, this is the first report demonstrating initiation of treatment for post‐HSCT complications based on app‐based digital monitoring. The SPIZ project is restricted by the geographic radius for home visits, as well as by patients' ability and willingness to use digital technology for symptom reporting and virtual visits. As the German healthcare system faces an aging population with increasing medical care needs, alongside a growing number of therapeutic options delivered in the outpatient setting, there is a clear need for innovative care structures outside of hospitals. Although final endpoint evaluation will be conducted after study completion, the preliminary results presented [42] indicate a high level of patient comfort in the intervention group. Findings from the SPIZ project and consecutive follow‐up studies may support the broader implementation of telemedicine infrastructures in cellular therapy follow‐up and facilitate the development of innovative outpatient care concepts for hemato‐oncological patients.

## Author Contributions

L.B. and V.V. wrote the manuscript. All authors contributed to the interpretation of the results and editing of the manuscript and agreed on the final version.

## Funding

The SPIZ study was supported by ‘Gemeinsamer Bundesausschuss’ of Germany (project number: 934500‐113, funding number: 01NVF22108).

## Ethics Statement

Written informed consent was obtained from the patient for the publication of any potentially identifiable images or data included in this article. Ethical approval is not required for this study in accordance with local or national guidelines.

## Conflicts of Interest

The authors declare no conflicts of interest.

## Data Availability

The data that support the findings of this study are available from the corresponding author upon reasonable request.
